# Detecting charge transfer at defects in 2D materials with electron ptychography

**DOI:** 10.1111/jmi.13404

**Published:** 2025-03-21

**Authors:** Christoph Hofer, Jacob Madsen, Toma Susi, Timothy J. Pennycook

**Affiliations:** ^1^ EMAT, University of Antwerp Antwerp Belgium; ^2^ Faculty of Physics, University of Vienna Vienna Austria

**Keywords:** bonding, density functional theory, scanning transmission electron microscopy, transition metal dichalcogenides, vacancies

## Abstract

Electronic charge transfer at the atomic scale can reveal fundamental information about chemical bonding, but is far more challenging to directly image than the atomic structure. The charge density is dominated by the atomic nuclei, with bonding causing only a small perturbation. Thus detecting any change due to bonding requires a higher level of sensitivity than imaging structure and the overall charge density. Here we achieve the sensitivity required to detect charge transfer in both pristine and defected monolayer WS_2_ using the high dose efficiency of electron ptychography and its ability to correct for lens aberrations. Excellent agreement is achieved with first‐principles image simulations including where thermal diffuse scattering is explicitly modelled via finite‐temperature molecular dynamics based on density functional theory. The focused‐probe ptychography configuration we use also provides the important ability to concurrently collect the annular dark‐field signal, which can be unambiguously interpreted in terms of the atomic structure and chemical identity of the atoms, independently of the charge transfer. Our results demonstrate both the power of ptychographic reconstructions and the importance of quantitatively accurate simulations to aid their interpretation.

## 1 INTRODUCTION

Chemical bonding makes a material with collective properties out of an ensemble of independent atoms. As a central tenet of density functional theory (DFT),^1^, ^2^ these properties can be entirely derived from the electron charge density. As such, measuring the charge density is of great interest, especially its variation from neutral independent atoms due to bonding. Electron diffraction and X‐ray scattering can detect charge transfer,^3^, ^4^, ^5^, ^6^ but both probe an average over a large area rather than individual atoms or atomic columns. For imaging atomic structures, electron microscopy has become an indispensable tool, and in recent years, scanning transmission electron microscopy (STEM) the de facto standard due to the simplicity of interpreting its annular dark‐field (ADF) atomic‐number contrast.^7^ However, ADF is only weakly sensitive to the electron charge distribution, as its contrast is dominated by Rutherford scattering from the nuclear cores. The inelastic scattering processes detected by electron energy‐loss spectroscopy (EELS) contain information about the local electronic environment^8^, ^9^ but require high electron irradiation doses as well as the use of especially demanding first‐principles spectrum simulations to interpret its fine structure.^8^

Phase‐contrast imaging methods, on the other hand, are known for being highly dose efficient, and indeed, local atomic‐scale detection of charge redistribution due to bonding has been demonstrated in high‐resolution transmission electron microscopy (HRTEM) for both single‐layer hexagonal boron nitride and doped graphene.^10^ However, revealing the charge redistribution in HRTEM required careful use of specific contrast transfer function (CTF) conditions with large defocus values, significantly degrading image resolution. Furthermore, due to the oscillatory CTFs of HRTEM, it is often difficult to interpret even for structural imaging, let alone disentangling charge transfer from that of the atomic structure. Fortunately, STEM phase‐contrast imaging has improved greatly in the past decade. Multiple phase‐imaging modes can be performed without the need for aberrations and in parallel with Z‐contrast ADF, which provides easily interpretable images of structures at the maximum resolution of the microscope. Atomic‐resolution charge‐density imaging has been performed with differential phase contrast (DPC), scattering center of mass (CoM)^11^ and off‐axis holography,^12^ but these techniques have not been sufficient to detect charge transfer due to bonding.

Here we use high‐speed^13^ focused‐probe single‐sideband (SSB) electron ptychography with post‐collection residual aberration correction^14^ to provide the necessary precision and accuracy to detect charge transfer directly in WS_2_, including at individual point defects. In addition to the high speed and dose‐efficiency of our method allowing us to outrun damage, the ptychographic aberration correction overcomes the problem of residual aberrations that has so far prevented CoM‐based methods from achieving valence imaging at atomic resolution.^15^, ^16^ Accounting for residual aberrations, as we do here, is essential because they can completely obscure the effect of charge transfer, and if ignored may result in an entirely incorrect interpretation. Also crucial is the use of our recent parameter‐based quantification method^17^ that explicitly accounts for the CTF of the imaging modality and overcomes artefacts due to the sample configuration such as mistilt.

Using the fact that the phase shift is directly proportional to the electric potential for thin samples, analogously to CoM imaging,^18^ we can take the Laplacian of the SSB phase image to obtain an image that is proportional to the charge density. This allows one to obtain an aberration‐corrected charge‐density map, which is not possible in present CoM‐ or DPC‐based methods. Here, we show that both ptychographic phase and charge‐density imaging can reveal the charge transfer, but analysing the phase images is more robust to noise than the charge‐density map itself because of its reliance on noise‐amplifying differentiation.^19^

## 2 RESULTS AND DISCUSSION

### 2.1 Pristine WS_2_

**FIGURE 1:**
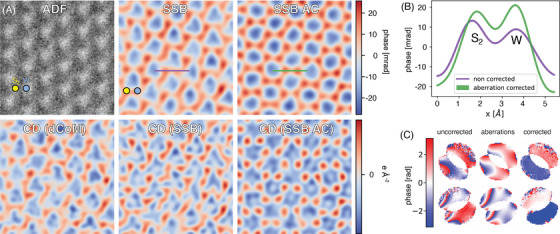
Residual aberration correction with ptychographic phase and charge‐density (CD) imaging of ${\mathrm{WS}}_{2}$. (A) Top row: Z‐contrast ADF, and simultaneous SSB images before and after residual aberration correction (AC). Bottom row: The charge density imaged using filtered conventional differentiated CoM (dCoM) and SSB ptychography before and after residual aberration correction. The dCoM image required Gaussian filtering to make the structure visible above the noise. Ptychographic residual aberration correction makes the SSB CD image significantly clearer than the dCoM and SSB CD images without it. (B) Line profiles from the SSB phase images in (A) showing how residual aberrations can reverse the polarity of the dumbbell, preventing the subtle effect of charge transfer from being detected without the ptychographic aberration correction. (C) Phase of the double‐disk overlaps at two different spatial frequencies, showing significant variation due to residual aberrations, which is much reduced upon correction.

The problem of residual aberrations is illustrated in Figure 1 with experimental data from WS_2_ using a modern aberration‐corrected STEM (see Methods in Appendix). Aberrations are particularly problematic when quantifying phase images since even small values can alter the phase.^20^ Two SSB images from the same dataset are shown in Figure 1A, one with ptychographic residual aberration correction and one without. Although the data were acquired while using a carefully tuned fifth‐order electron‐optical aberration corrector, the lattice polarity in the SSB image without the additional ptychographic post‐collection correction is reversed because of the residual aberrations, as illustrated by the line profiles in Figure 1B. With such large effects on the phase images resulting from such relatively small aberrations, reliably interpreting uncorrected phase images from either ptychography‐ or CoM‐based methods, including DPC, to uncover the subtle effects of charge transfer is in our view essentially untenable.

For very thin specimens such as 2D materials, the phase without aberrations should be flat in the double‐disk overlaps in probe reciprocal space. The double‐disk overlaps from two different spatial frequencies contained in the data are shown in Figure 1C. Without ptychographic aberration correction, the double‐disk overlaps of both spatial frequencies are not flat. We use singular value decomposition (SVD) to identify the aberrations present and counteract the phases of these aberrations, resulting in a nearly flat phase in each of the overlap regions, confirming the removal of the residual aberrations.^14^ See Supplementary Material for additional information.

Figure 1A also shows charge‐density maps calculated from the CoM and from the ptychographic phase images using differentiation. The raw CoM‐based charge‐density map, the differentiated CoM (dCoM), is very noisy indeed and a Gaussian filter is required to make the structure apparent over the severe noise (see Figure S2). The resulting filtered dCoM image shown in Figure 1A is fairly similar to the SSB‐based charge‐density map without ptychographic aberration correction. The SSB‐based charge‐density map with the additional ptychographic aberration correction, however, shows a much higher‐quality image. As we will show, our post‐collection aberration‐corrected ptychographic phase images and charge‐density maps show an excellent match with simulations that include charge transfer.

**FIGURE 2:**
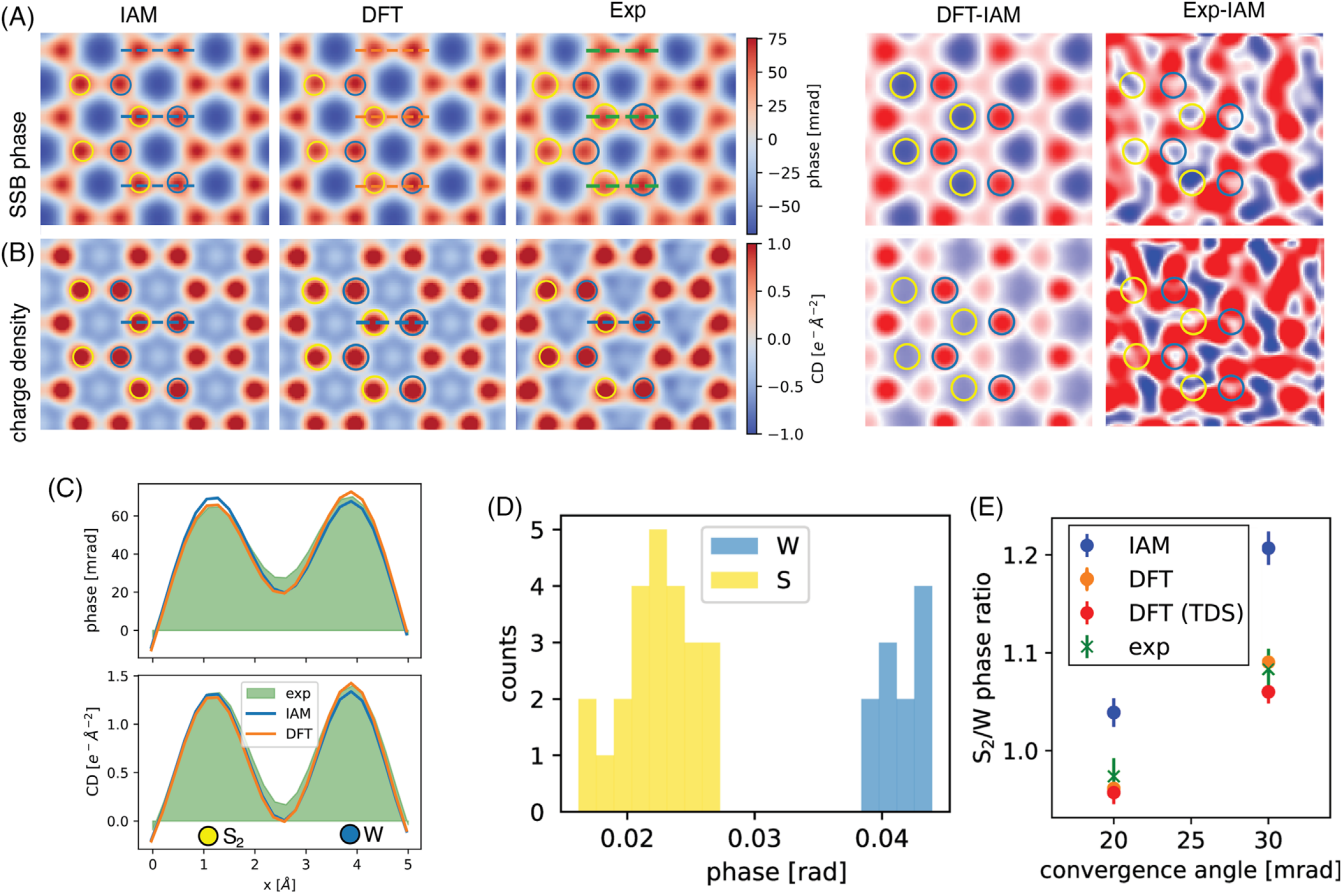
Detecting charge transfer in WS_2_. (A) SSB phase images reconstructed from simulated and experimental data of pristine WS_2_. The experimental data is averaged over the area shown in Figure S2. The difference between the DFT and IAM phase images results from the electron transfer from W to S. The experimental difference to the IAM‐based simulations shows a qualitative match with the theoretical difference. (B) Charge‐density images based on the SSB phase. The charge transfer is visible in the difference image, but the experimental difference image is noisier than the SSB phase‐difference image. (C) Line profiles of the experimental and simulated images showing an excellent match between DFT and experiment despite the small difference between the IAM and DFT. (D) Histogram of deconvolved S and W phases in the 30 mrad experimental SSB image. (E) Ratios of the mean values of the two sublattices using IAM and DFT potentials with and without the effect of thermal diffuse scattering (TDS) compared to experimental data for 20 and 30 mrad convergence angles.

To understand the role of charge transfer in the observed contrast, we simulated images with and without the effects of bonding. To do so, we used potentials based on first‐principles DFT and the independent atom model (IAM) as input to multislice 4D‐STEM simulations using the *ab*TEM code,^21^ with probe parameters and sampling set to match our experimental conditions (see Methods in Appendix). Figure 2 shows the ptychographic phase and charge‐density images simulated based on the DFT and IAM potentials. The figure also shows the differences between the DFT‐ and IAM‐based images, that is, the quantitative difference induced by the bonding. Compared to the IAM the DFT phase image has a higher phase on the W sites and a reduced phase on the $S_{2}$ sites. Compared to the IAM‐based ptychographic charge‐density image, the DFT‐based result shows a higher charge density at the W sites and a reduced charge density at the $S_{2}$ sites.

As the charge redistribution due to bonding contained in the DFT potential is the only significant difference between the DFT and IAM images, the differences between them are attributed to the charge transfer from W to S that occurs with electron redistribution due to bonding (see also Figures S3 and S4). Importantly, the magnitude of the difference due to bonding is around 10% of the maximum values at the atomic sites in the DFT and IAM images, enough to be detected at moderate electron doses (see Supplementary Material).

Figure 2 also shows the result of subtracting the IAM phase and charge‐density images from the experimental images. Note that the experimental images shown in this figure are area‐averages from the area shown in Figure S2, and that the theoretical images are scaled to match the mean and standard deviation of the experimental images. The subtraction is very difficult and prone to error, especially because small mismatches in the alignment of the images will result in large values in the difference images. Despite the difficulty, a qualitative match with the theoretical image is obtained. The experimental difference image does however exhibit a magnitude three times higher than the theoretical difference, especially closer to the center of the hexagons. We expect this difference is mostly due to misalignment caused by experimental scan distortions, sample drift and mistilt; however, residual aberrations which are not perfectly accounted for even with post‐collection aberration correction may also contribute.

The phase differences between the IAM, DFT and experimental results are also reflected in the line profiles taken across the W‐$S_{2}$ dumbbells shown in Figure 2C. An average of 45 dumbbells was used for the experimental profiles to provide high statistical significance. Interestingly, the charge‐density image provides less statistical significance than the phase image, as the difference image is noisier. This is likely because calculating the charge‐density map involves double differentiation, and differentiation is often associated with noise amplification. We therefore propose that analysing the phase is more reliable when it comes to charge transfer detection.

To quantify the influence of charge redistribution from the ptychographic images, we need to assign a phase to each of the atomic sites. Various approaches have been established for quantifying the atomic intensities of ADF images, including local maxima,^7^ the integration of Voronoi cells,^22^ Gaussian fits,^23^, ^24^ as well as template matching.^25^ However, these quantification methods do not work well for methods with low transfer of low spatial frequencies such as ptychography and filtered CoM‐based imaging, as they induce a nonlinear dependence on the local atomic environment:^17^ the proximity of neighbouring atoms can actually decrease the intensity at which they appear in images, which is very different to the additive behaviour of probe tails in ADF imaging. This introduces a strong image dependence not only on the atomic arrangement but also on sample tilt, which must be accounted for to correctly quantify such phase images, but is not accounted for in any of the usual image quantification methods designed for ADF imaging.

To overcome this problem, we recently developed a quantification method that specifically accounts for the contrast transfer function (CTF) of the imaging method and is robust to both sample tilt and source size.^17^ A model fitting the experimental data is initiated and a fast simulation using the convolution between the point‐potential of the model and a kernel representing the CTF is optimised with respect to the experimental image. This is especially important here because mistilt is particularly difficult to eliminate in 2D materials and would cause significant errors in quantification, in addition to any uncorrected residual aberrations. With this method, the quantified phase of an atomic column is independent of both the proximity and type of the neighbouring atoms. In addition to the SSB used presently, the quantification method can also be used for iterative ptychography and filtered CoM‐based imaging, which can also exhibit a negative halo around isolated atoms, as we have demonstrated.^17^

Figure 2D shows a histogram of the quantified phases of the W and S atoms in the experimental image. The phases of the two types of atoms are well separated. The ratio of the mean quantified phase values of $S_{2}$ to W depends on the convergence angle as shown in Figure 2E and, crucially, matches the results of the DFT simulations and not the IAM results.

The variation with the convergence angle occurs because it determines the strength at which different spatial frequencies are transferred.^26^, ^27^ This in turn determines the relative strengths at which the features of the charge‐density distribution appear in the phase images. The excellent agreement of the experiment at both convergence angles with the DFT results, and lack thereof for the IAM results, demonstrates that the frequencies transferred by these conditions contain the information on the charge transfer induced by bonding. We emphasise that such conditions are typical for high‐resolution STEM imaging. This is in contrast to the situation in conventional HRTEM, where the imaging conditions relevant to detecting charge transfer are generally not those desired for imaging the structure itself. Including atomic vibrations using thermal diffuse scattering (TDS, see Methods in Appendix) slightly decreases the S_2_ to W ratio compared to neglecting it, and the experimental uncertainty, estimated from the statistical site‐by‐site phase variation, dominated by the limited signal, overlaps both values with bonding included. The ability to discern the charge transfer of course depends on the noise level imposed by the dose and the sample size as discussed in the Supplementary Material.

### 2.2 Defective WS_2_

**FIGURE 3:**
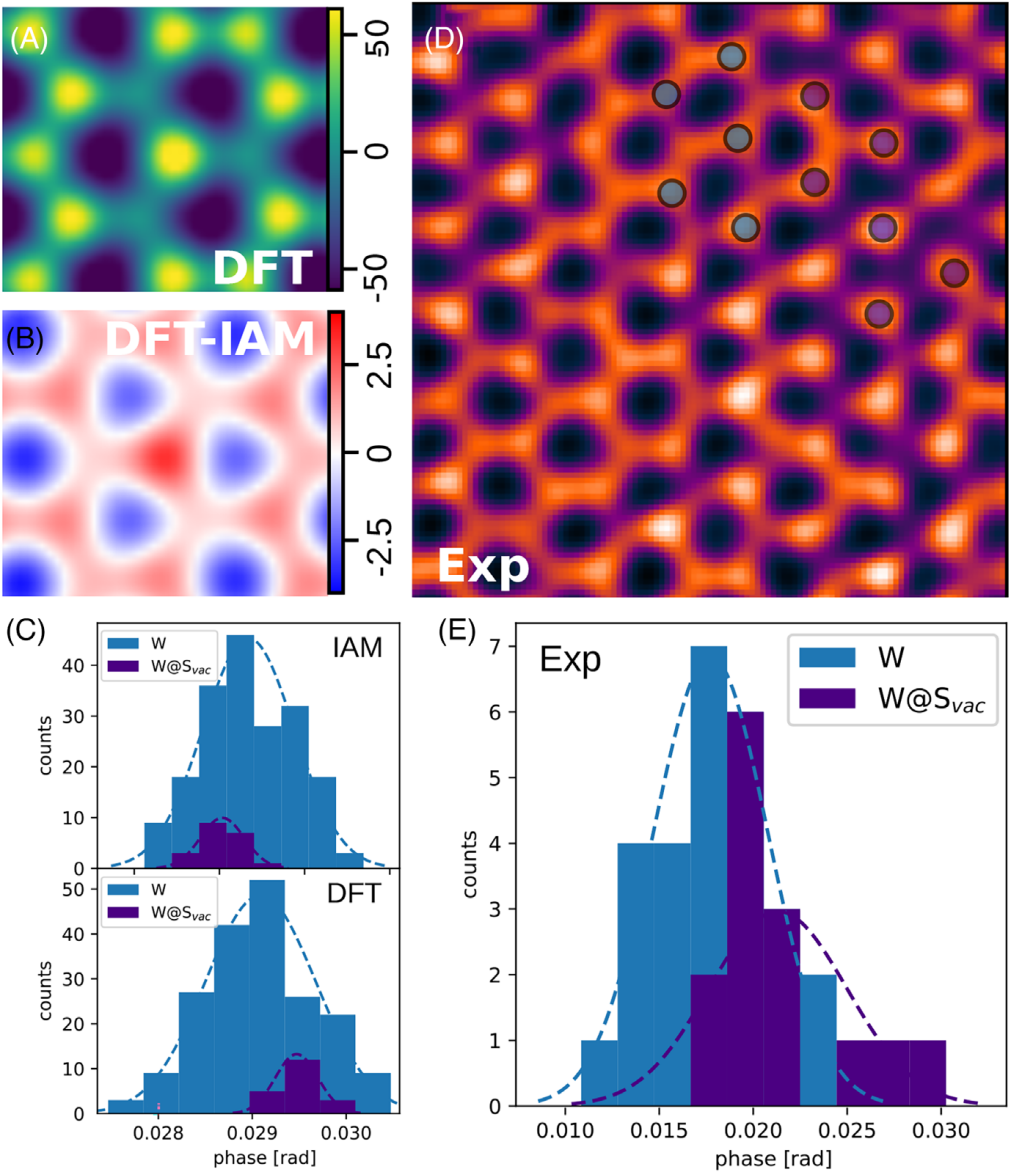
Analysis of defective WS_2_. (A) SSB reconstruction based on a DFT potential of WS_2_ with three S vacancies distributed about the central W atom. (B) Difference between IAM‐ and DFT‐based simulations of the same structure. (C) Histograms of extracted deconvolved phases of IAM‐ and DFT‐based simulations of a defective area similar to the experimental defect density, showing a difference between W in the pristine (blue) and the high defect‐density region (dark blue) at a $1\times 10^{5}$ $e^{-}/$Å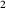 dose. The statistical distribution is based on 10 randomly noised 4D data sets with this dose. (D) Experimental SSB image of defective WS_2_. The overlaid circles indicate pristine W (blue) and W close to the defects (dark blue). (E) Histogram of the deconvolved phases of pristine W (blue) and W surrounded by vacancies (dark blue) extracted from the image in (D).

The simplest defect in transition metal dichalcogenides is a chalcogen vacancy. For WS_2_ such a monovacancy ($V_{S}$) can be realised by removing a single S atom, leaving both a S vacancy and one remaining S atom at the former S_2_ site. Such vacancies produce a significantly reduced phase at the vacancy sites in SSB reconstructions, as shown in the DFT‐based SSB image in Figure 3A, where three S monovacancies ($V_{3S}$) are distributed around the central W atom. Note that the S sites are in general not readily visible in the high‐angle ADF signal under these conditions, and thus S vacancies are only discernible in the phase images (see also Figures S7 and S8), highlighting the ability of ptychography to reveal light atoms next to heavy elements.^14^, ^28^

Interestingly, we observe a higher W site phase close to the S vacancies in our SSB images, with the intensity of the W depending on the density of the vacancies in the area. The reason for this is twofold: the CTF and the charge transfer itself. As we discussed earlier, the SSB CTF means that the phase of atoms is reduced in SSB images when they are brought near to each other. Therefore, removing an atom to form a vacancy significantly increases the phase of the neighbouring sites in SSB images even for neutral atoms. It is therefore vital to take this CTF effect into account, as we do with our method of phase quantification, allowing us to detect the second effect, the charge transfer.

To systematically study the charge transfer at defects, we simulated images of a region of WS_2_ with increasing numbers of S vacancies using both IAM and DFT potentials (Figure S9). For the the $V_{3S}$ configuration shown in Figure 3A, the difference between the IAM‐ and DFT‐based image simulations is displayed in Figure 3B. Keeping in mind the differences seen in pristine WS_2_, the effect of the vacancies on the difference is greatest at the W atom surrounded by the vacancies. Furthermore, we find that the phase of a W atom in WS_2_ increases in the DFT‐based images relative to that of the IAM‐based images proportionally with the density of S vacancies around the W atom. This is clearly seen in Figure S9, with the DFT phase at the W atom increasing significantly more with increasing vacancy density than the IAM.

**FIGURE 4:**
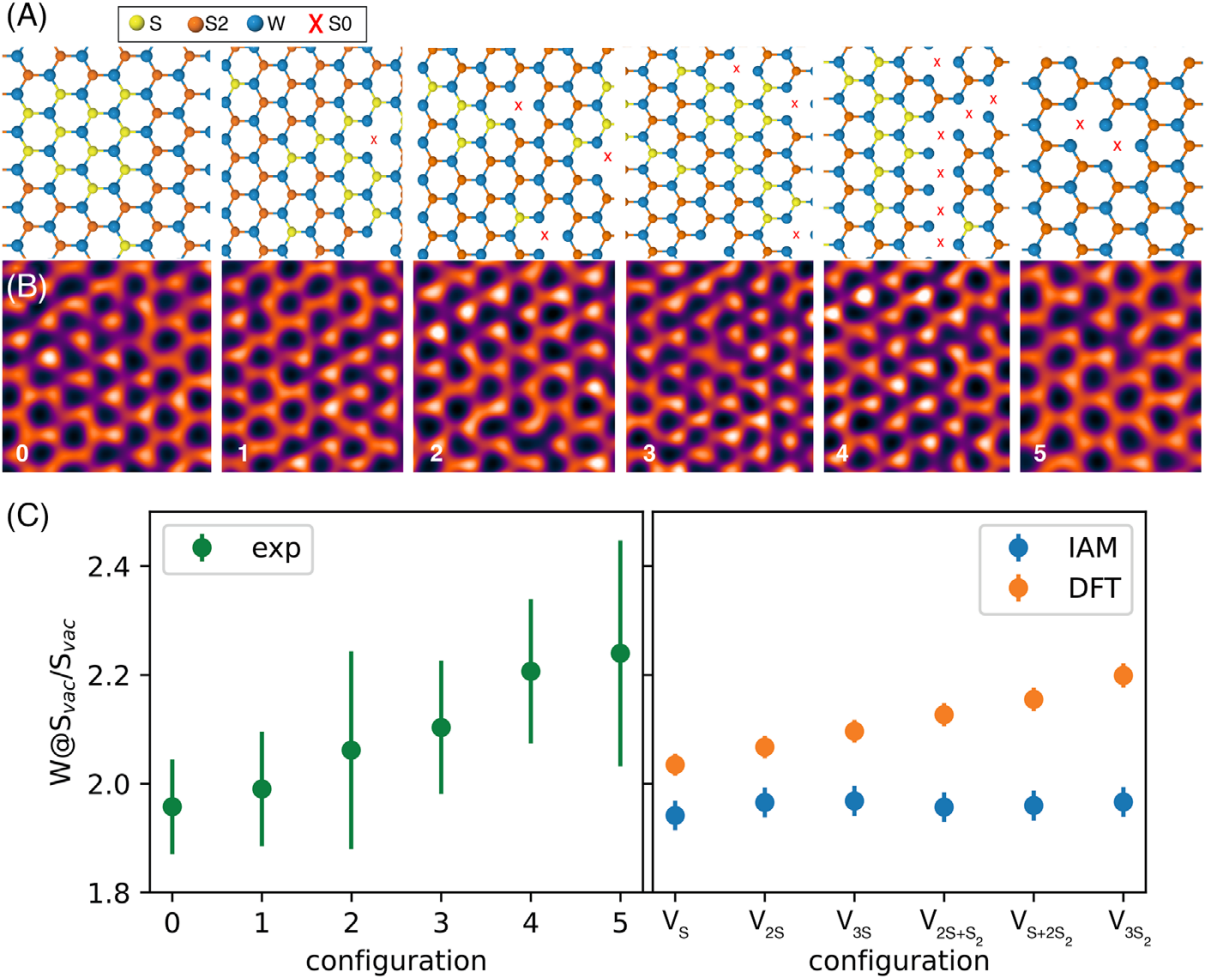
Analysis of the dependence of the $W@S_{\mathrm{vac}}/S_{\mathrm{vac}}$ phase ratio on defect density. (A, B) Atomic models and SSB images of defective WS_2_ containing different numbers of mono‐ and divacancies. (C) Phase ratio between W atoms next to vacancies and at S monovacancies. The density of vacancies around the $W@S_{\mathrm{vac}}/S_{\mathrm{vac}}$ increases from left to right for both the experimental (left) and simulated (right) data. The ratio increases with vacancy density for both the experimental data and the DFT‐based simulations containing charge transfer due to bonding, whereas the IAM simulations excluding bonding do not.

The variation of the quantified phase ratio of the central W site to a S vacancy site ($W@S_{\mathrm{vac}}/S_{\mathrm{vac}}$) with respect to the density of vacancies for the DFT‐ and IAM‐based simulations is shown in Figure 4C. Each subsequent configuration in the simulations has one less S atom. The ratios for the IAM simulation results are essentially flat, while the DFT ratios increase linearly with the density of vacancies. The flatness of the IAM result is exactly what is expected given our quantification method accounts for changes that are purely structural. As the only significant difference between the IAM and DFT images is the charge transfer in the DFT‐based images, the DFT results show that the amount of charge transfer at a W site increases with the amount of vacancies surrounding it and that this results in a higher quantified phase of the site.

This increase in the charge transfer with vacancy density would be surprising if one assumes that vacancies reduce charge transfer due to the W atoms having fewer S neighbours. If that were the case, one would see a reduced phase in the DFT images relative to the the IAM images as the vacancy density is increased, rather than the observed increase. In the extreme three‐divacancy configuration ($V_{3S_{2}}$), the W atom has lost all its nearest neighbours and one might think is therefore more similar to an independent atom and expect a smaller difference between the DFT and IAM results. However, this is clearly not the case, as seen in the simulated SSB images and projected all‐electron potentials shown in Figures S9 and S10, and this highly defected case is in fact the furthest from the IAM result with a higher $W@S_{\mathrm{vac}}/S_{\mathrm{vac}}$ ratio than all the other results using the DFT potentials. The fact that S vacancies produce occupied states at the defect sites^29^, ^30^ does not explain the observed contrast, and additional studies have shown charge redistribution occurs towards the defect sites.^31^

To understand this physical phenomenon, we plot the three‐dimensional (pseudo‐)electron valence densities of each configuration in Figure S11, from which a different picture emerges. The line profiles from W sites adjacent to the defects show a lower density of valence electrons when increasing the number of vacancies around them, in agreement with the increase of phase we observe at this site. The electrons are redistributed towards the next W sites, which creates W‐W bonding that is stronger the more S vacancies are involved in the system. Thus we need to consider longer‐range charge rearrangement than merely the first‐nearest neighbours to understand the full picture.

The relative difference between IAM and DFT phases can reach up to 10% with vacancies (cf. Figure 4C). This is a larger difference than in the pristine case, and one might thus conclude that detecting charge transfer is easier at defects. However, defect configurations are often unique and complex and therefore a statistical assessment analysing multiple sites is very challenging with each atom potentially undergoing different amounts of charge transfer. Furthermore, the dose one can use to probe defective configurations is usually lower due to their greater propensity to damage, increasing the uncertainty per site. In this case the dose we could use before damage was only $3\times 10^{4}$ $e^{-}/$Å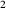.

To ascertain whether we can detect charge transfer at defects experimentally, we used electron irradiation to obtain a variety of vacancy densities and atomic configurations in the sample. A representative area is shown in Figure 3D, with the full field of view shown in Figure S7. Figure 3C shows histograms of the extracted phases of the W atoms in the pristine area (blue) and the W atoms adjacent to vacancies, labelled as $W@S_{\mathrm{vac}}$ (dark purple) for the IAM‐ and DFT‐based simulations. Both types of simulations were treated with Poisson noise in the 4D diffraction data corresponding to the experimental dose. The calculated mean value for $W@S_{\mathrm{vac}}$ shows a significant increase in phase compared to pristine W atoms in the DFT simulations. Note that the structure analysed here only contains monovacancies (cf. Figure S8a and b) and that the phase shift due to charge transfer would be significantly higher if this specific area of interest also contained divacancies. This agrees with the experimental data, where an increase in the mean phase of the $W@S_{\mathrm{vac}}$ compared to that of the pristine W sites is indeed observable in Figure 3E.

To further study the effect of the vacancies on the charge transfer experimentally, we calculated the phase ratio of the sites between which the majority of the charge transfer occurs, $W@S_{\mathrm{vac}}/S_{\mathrm{vac}}$, using the five different regions of the sample shown in SSB images in Figure 4B. Each region has a different vacancy configuration, labelled from zero to five, and a model for each is shown in Figure 4A. The experimental ratios are shown in Figure 4C alongside the values calculated from the DFT‐ and IAM‐based simulations. Both experimental and DFT configurations are presented in their respective plots in order of increasing vacancy density around the measured $W@S_{\mathrm{vac}}$. Of the experimental configurations, zero has the lowest average number of vacancies around the $W@S_{\mathrm{vac}}$ and five has the highest.

The low doses used in the experiment produce significant error bars particularly for the experimental configurations with very few $W@S_{\mathrm{vac}}$, such as configuration five with only five $W@S_{\mathrm{vac}}$. However the overall effect seems clear: as the density of vacancies around the $W@S_{\mathrm{vac}}$ increases so too does the ratio $W@S_{\mathrm{vac}}/S_{\mathrm{vac}}$, entirely consistent with the findings of our theoretical calculations. Experimental configuration five contains only double vacancies, and they are next to each other, meaning the $W@S_{\mathrm{vac}}$ are either next to one or two double vacancies. The calculated ratio for this configuration is the highest, consistent with those of the DFT configurations containing divacancies. Experimental configuration zero, on the other hand, contains no divacancies and has a much lower ratio, more consistent with the DFT calculations containing only monovacancies.

Clearly we are hindered in our ability to quantify the charge transfer itself in our experiments with defects at the level of precision imposed by the dose required here to avoid damaging the defects, as well as the small sample sizes used. Furthermore the experimental structures contain multiple types of vacancy configurations and are generally part of a larger, more complex structural environment than captured by our DFT calculations. Nonetheless, the increase in the mean experimental ratios with the density of vacancies at the measured sites fits very well with those predicted from theory. It therefore seems clear we can indeed detect the charge transfer caused by defects and indeed are sensitive to their density.

## 3 CONCLUSIONS

To conclude, we have experimentally detected charge transfer in monolayer WS_2_ including at its defects via the influence of the electron distribution in the material on the phase of the probe electrons using electron ptychography and a parameter‐based quantification method. Post‐collection aberration correction is crucial to obtain accurate phases for charge‐transfer measurements, and the convergence angle is also an important parameter, as it defines which spatial frequencies are transferred. Our results indicate that the frequencies at which charge transfer is detectable conveniently include those used for atomic‐resolution imaging. Simulations based on first‐principles charge redistribution due to bonding lead to an excellent agreement in the pristine case. For defective areas, we observe a notable phase increase of the metal sites close to chalcogen vacancy sites which significantly increases with the density of vacancies involved, consistent with our theoretical results including charge transfer due to bonding. Our study thus presents a significant advance in detecting atomic‐scale charge transfer at defects.

## Supporting information

Supporting Information

## METHODS

### A.1 Sample preparation

WS_2_ was synthesized on Si/SiO_2_ by physical vapor deposition from WS_2_ powder. The flakes were then transferred to a Quantifoil(R) TEM grid by a drop of isopropanol. The transfer was completed by etching the SiO_2_ with a KOH solution and cleaning by deionized water.

### A.2 Transmission electron microscopy

STEM experiments were conducted on a ThermoFisher Themis Z instrument equipped with a Timepix3 camera at 60 keV. The probe convergence angle was set to 30 mrad unless noted otherwise, and the beam current was ca. 2 pA. Typical 4D datasets included 1024 × 1024 probe positions with a dwell time of 1–5 μs. The list of events provided by the Timepix3 was converted to 4D data using an in‐house code. The detector sampling was chosen to be approx. 30–40 pixels for the bright field disk and the real‐space sampling was ca. 0.1–0.15 Å per pixel. The ADF detector semi‐angular range was 70 to 220 mrad. To increase the signal‐to‐noise ratio, several frames (up to 10) were aligned and averaged. Typical resulting total doses were approx. $10^{5}$ $e^{-}/$Å.

### A.3 Density functional theory

For DFT, we used the projector‐augmented wave method in the open‐source package GPAW.^32^ The hexagonal unit cell of ${\mathrm{WS}}_{2}$ was made orthogonal, its size was optimized, and then used to create 5×3×1 supercells of ${\mathrm{WS}}_{2}$ (a total of 90 atoms) with 8.9 Å of vacuum between the periodic images of the layers (perpendicular cell size of 12 Å). To obtain relaxed structures for the multislice simulations, the atomic positions were optimized using a plane‐wave basis with a cutoff energy of 500 eV and 3×3×1 **k**‐points to sample the Brillouin zone until residual forces were below 0.02 eV/Å. To create models with defects, we removed one or more S atoms, and rerelaxed the atomic positions.

To verify the convergence of the computational parameters, we further relaxed selected models (pristine, 3MV, 3DV) using a higher plane‐wave cutoff of 800 eV, a denser **k**‐point mesh of 5×5×1, and included spin polarization. For these DFT potentials, we ran the full 4D‐STEM simulations followed by the single‐sideband reconstruction and kernel optimization (see below). Differences in the phase ratios of the sites with respect to the default parameters were at most 0.5%, which we have included in the uncertainty estimates for our computational values.

Charge transfer between the W and $S_{2}$ columns was estimated via Bader partitioning^33^ of the all‐electron charge density using the grid‐based implementation of Henkelman and colleagues.^34^ The resulting atomic charges for the pristine system were compared to those calculated for atoms surrounding the S vacancy.

### A.4 Multislice simulations

For the multislice simulations, the DFT potential was calculated from the all‐electron charge density converged with GPAW, as described previously.^35^ For the IAM, the potential was taken to be a superposition of individual tabulated atomic potentials, as parametrized by Lobato.^36^ A lateral real‐space sampling of 0.05 Å has been used for both potentials as well as a perpendicular slice thickness of 1.0 Å.

Thermal diffuse scattering was included by running DFT‐based velocity‐Verlet molecular dynamics (MD) with a timestep of 2 fs, initialized with a Maxwell–Boltzmann temperature of 300 K and thermalized for 100 timesteps. Ten MD frozen‐phonon snapshots were generated by saving the converged electron density every 50 timesteps, and datasets averaged over the ensemble. Although more images would likely be required for full convergence of an atomically thin material, the cost of DFT/MD for large supercells is high and considering the small effect of TDS on the phase ratios, this does not affect our conclusions.

4D‐STEM simulations were carried out using the multislice approach as implemented in the open‐source package *ab*TEM.^21^ All simulation parameters were set to the experimental parameters, including the convergence angles and the electron dose per area simulated as Poisson noise of the diffraction patterns. To estimate statistical variation caused by the limited dose, multiple randomly noised datasets were analysed as indicated in the main text. The reciprocal‐space sampling was 0.06 Å. For ADF, we note that DFT and IAM potentials produce essentially identical contrast.

### A.5 Single‐sideband ptychography

SSB ptychography was performed with the open‐source PyPtychoSTEM package,^37^ using either the experimental or simulated 4D datasets as input. The step size was 0.1–0.15 Å per pixel depending on the dataset and the voltage was 60 kV. The convergence angle was set to 20 or 30 mrad. Post‐collection aberration correction was applied using singular value decomposition to identify the residual aberrations which were then counteracted. More details are given in Supplementary Material.

### A.6 Kernel method for assigning phases to atomic sites

The phases were extracted by an optimization method where atomic phases are fitted to the target image.^17^ First, an initial atomic model that represents the experimental configuration is aligned with the target image. The model is converted to a point potential, which is convoluted with a kernel calculated by the contrast transfer function of SSB. The resulting simulation is compared with the target image and their correlation is maximized by optimizing the alignment between the model and the experimental image, the width of the kernel and the intensities of the point potential. For the experimental data, the sample tilt is also optimized. Correlations were above 99% for the simulated data and above 93% for the experimental data.
