# Supplementary material for: Detecting charge transfer at defects in 2D materials with electron ptychography
Source: J Microsc. 2025 Mar 21;300(2):156–66. doi: 10.1111/jmi.13404 (PMC12523977; doi:10.1111/jmi.13404)
Supplement: Supplementary file 1 — Supporting Information [file JMI-300-156-s001.pdf]

Supplementary Material

S1. Post-acquisition aberration correction

Correcting the residual aberrations in phase images is crucial to determine the phase changes imposed by the sample accurately. In SSB, the double-disk overlaps in probe reciprocal space can be used to analyze any aberrations present. SFig. 1 shows examples of the double-disk overlaps from one of our datasets for four different spatial frequencies. The top row shows the experimental phases, the middle row the calculated phases with the identified aberrations and the bottom row the compensated phases, which are much more flat. The phase of the double-disk overlaps should be flat in the absence of aberrations. The residual aberrations identified by singular value decomposition (SVD) in the data shown in Fig. 1 are listed in Table 1. Note that we used a fifth-order electron-optical aberration corrector in the experiments, which balances the aberrations up to fifth order to oppose the uncorrected seventh-order aberrations. The SVD algorithm in contrast tries to fit the phase using aberrations up to the order of coefficients it is told to use. This can result in different values than what will be reported by the tuning software of the hardware aberration corrector.

S2. dCoM based charge density maps

SFig. 2 illustrates the benefits of ptychographic phase imaging over direct charge-density imaging based on the CoM data. The dCoM charge-density map is not only affected by aberrations, but is also far noisier at a given dose. A Gaussian filter reveals the atomic structure, but the strong variation over the atomic sites in the filtered images is still clearly too high to quantify the charge transfer. The ptychographic phase image with post-collection aberration correction on the other hand is far clearer and sufficiently sensitive to detect the charge transfer.

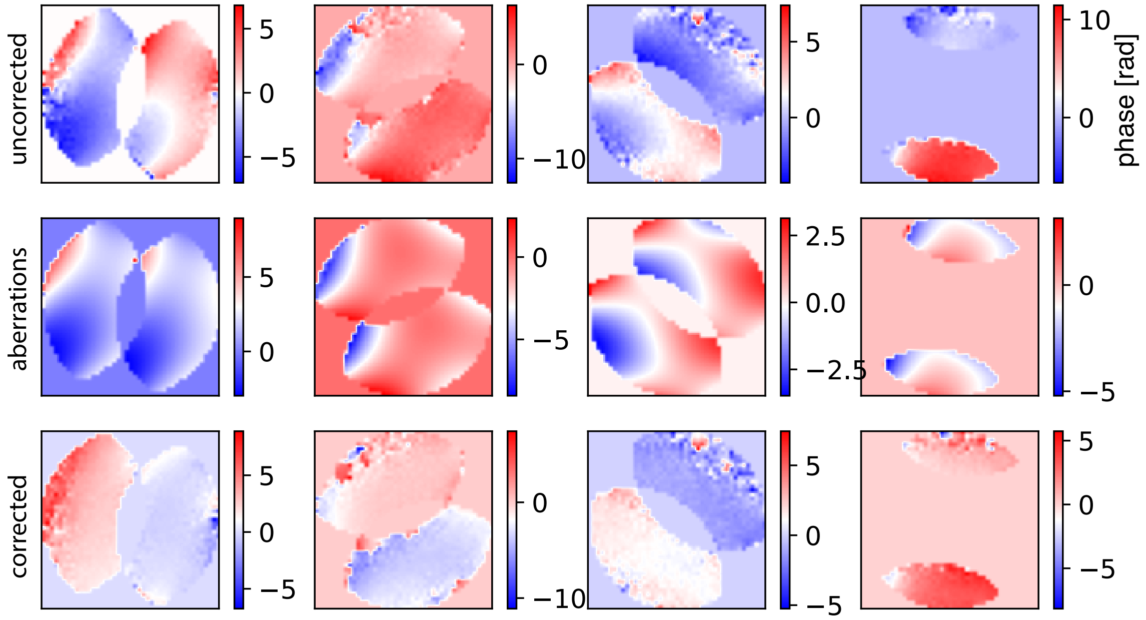

Supplementary Figure 1. Unwrapped phases of the double-disk overlaps (DDOs) at different frequencies. Top: Uncorrected DDO phases. Middle: SVD-calculated DDO phases used for the aberration correction. Bottom: Compensated DDO phases.

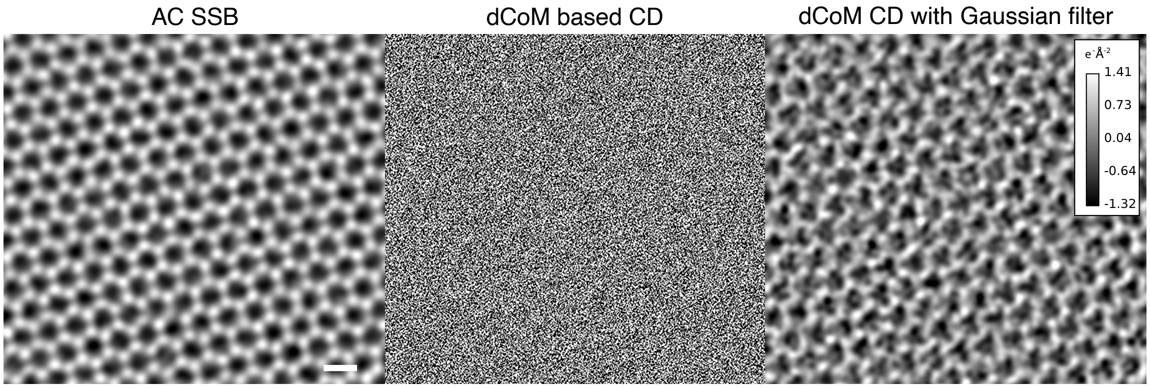

Supplementary Figure 2. SSB phase image and dCoM based charge-density (CD) maps of pristine WS_2. The dose is approximately 5×10^4 e$^-$Å^{-2}. The left image shows the SSB image with ptychographic aberration-correction (AC). The middle image is the raw dCoM based charge-density map, which is very noisy due to the limited amount of electrons. Gaussian filtering reveals the atomic structure (right).

Supplementary Table 1. Aberration coefficients up to 5th order of the experimental data shown in Fig. 1 of the main text as determined by the SVD algorithm.

C10	3.923 nm
C12a	4.494 nm
C12b	0.719 nm
C21a	425.722 nm
C21b	-538.706 nm
C23a	-29.749 nm
C23b	-173.962 nm
C30	-18.358 μm
C32a	-23.239 μm
C32b	-30.227 μm
C34a	2.782 μm
C34b	-0.213 μm
C41a	-0.491 mm
C41b	0.322 mm
C43a	-0.292 mm
C43b	0.369 mm
C45a	-0.083 mm
C45b	-0.134 mm
C50	7.208 mm
C52a	24.914 mm
C52b	34.317 mm
C54a	-9.11 mm
C54b	10.464 mm
C56a	-5.105 mm
C56b	8.305 mm

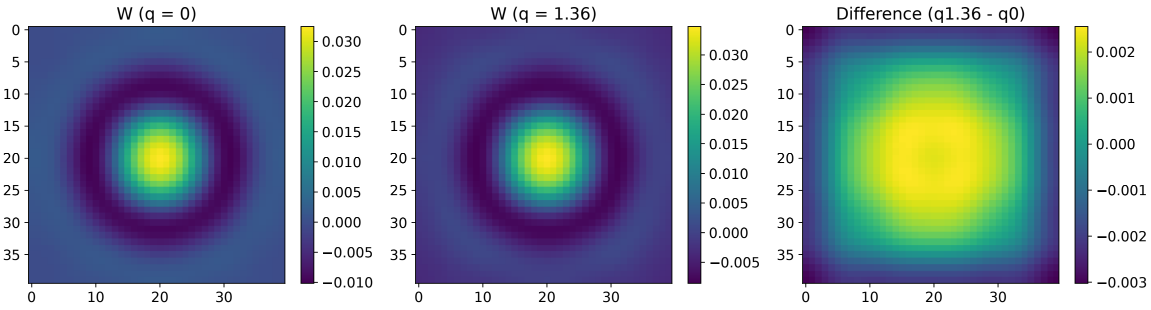

Supplementary Figure 3. Phase change induced by charge removal. Left: Phase image of an independent neutral W atom. Middle: Phase image of a W atom with a removed charge of 1.36 electrons. Right: Difference between the two. The negative values at the edge are due to the finite size of the cell and the uniform compensating background charge applied to the charged system.

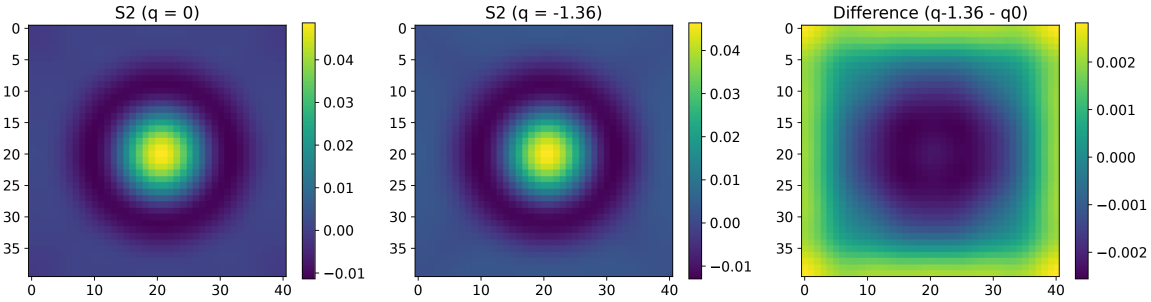

Supplementary Figure 4. Phase change induced by charge addition. Left: Phase image of an isolated neutral S_2 column. Middle: Phase image of a S_2 column with an added charge of 1.36 electrons. Right: Difference between the two. The positive values at the edge are due to the finite size of the cell and the uniform compensating background charge applied to the charged system.

S3. Effect of charge transfer on phase shifts

In pristine WS_2, the less electronegative W atoms donate 1.36 electrons to the neighboring S. Using DFT potentials for W atom SSB ptychography simulations, we can study the effect of charge removal or addition on the SSB phase, as shown in SFigs. 3 and 4. The left panels show an isolated W atom or S_2 column, which corresponds to an IAM model. The middle panels show the phase for these models with an additional / reduced charge q equal to 1.36 elementary charges, i.e. to a removal / addition of 1.36 electrons, corresponding to the charge transfer in WS_2. As indicated by the difference between the two plotted in the right columns, the phase change is up to 8% for the W and -5% for S_2. In practice, the differences are smaller as charge is not completely removed into a uniform compensating background charge as required by DFT, but rather transferred to nearby sites. This example corresponds

to perfect ionicity of the chemical bond, while in practice any covalency reduces the contrast. The crucial point, however, is that due to its effect on screening the nucleus, charge transfer can result in an order of magnitude larger change in the phase than in the potential itself, which only changes by 0.5%. This larger change in the phase substantially aids the detection of charge transfer.

S4. IAM parametrization

Another potential source of uncertainty in our analysis of the simulated phase ratios are unavoidable differences in how the IAM and DFT potentials are constructed. From GPAW, we recover the exact core-electron density from the projector augmentation functions, to which the self-consistent valence density is added alongside the nuclear contributions. An IAM such as the Lobato parametrization, on the other hand, is a fit to an atomic radial potential typically obtained from a relativistic Hartree-Fock calculation, with a limited number of fitting parameters and a specific functional form. Although care has been taken in *ab*TEM to ensure that DFT and IAM potentials are constructed in a consistent way, phase differences due to charge transfer are so small that minor numerical details might matter.

To estimate this effect, we ran GPAW calculations for isolated neutral W and S atoms, and fitted the resulting radial potentials with the parametrization used by Lobato to create a custom IAM based on our DFT potential. SFig. 5 shows the fitted potential compared to the original Lobato parametrization, with only very minor differences further away from the nucleus. We then performed our full analysis (generation of potentials, 4D-STEM simulation, SSB reconstruction, kernel optimization) on isolated W and S_2 columns using both potentials, and found a 0.9% difference in the S_2/W phase ratio between the two parametrizations. Although our full DFT potential may arguably be more accurate, we have nonetheless included this as an uncertainty for our simulated IAM values in the main text.

S5. Phase uncertainty due to limited dose

Experimentally, sufficient precision is either achieved with high doses, combining measurements from multiple identical atomic sites, or both. Dose is the most limiting factor when it comes to phase-difference measurements, thus averaging over multiple sites is often unavoidable. The amount of electron dose a specimen can handle until damage occurs depends very much on the material and typical doses for imaging 2D materials range from 10^4–10^6 e^-Å^{-2}. The difference between the S_2/W ratios have to be sufficiently separated so that their uncertainties are not overlapping. The error bars, which are calculated by the variation of phases due to noise, depend on the dose as well as on the number of atoms analysed. Thus obtaining sufficient statistical precision for configurations with small numbers of atoms is much more difficult.

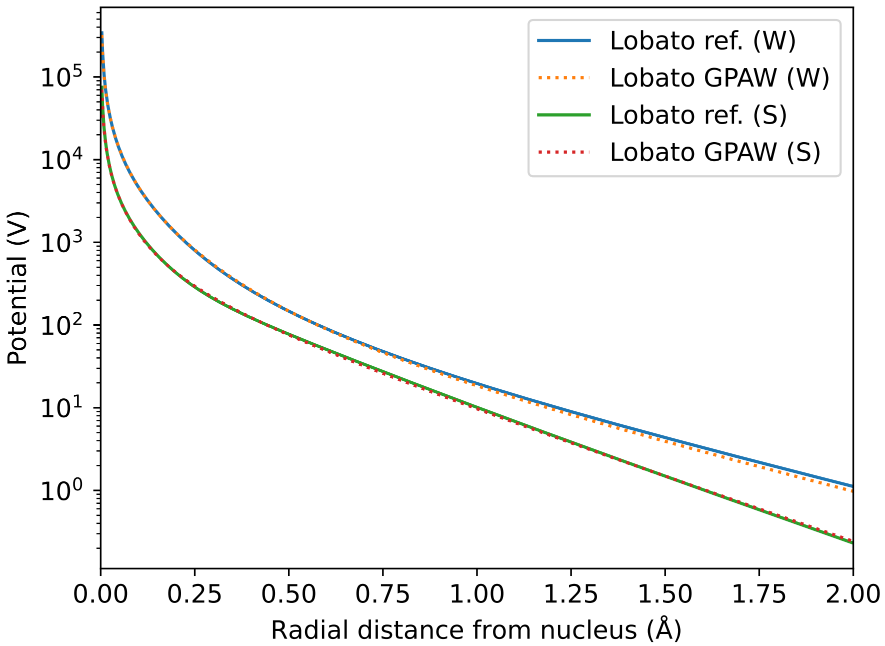

Supplementary Figure 5. Comparing IAM parametrizations. Radial potentials for W and S atoms based on the IAM parametrization of Lobato (solid lines) compared to a custom parametrization of the same functional form fitted to an atomic GPAW calculation (dotted lines). Minor differences can be observed further away from the nuclei (note the logarithmic scale of the vertical axis).

To estimate the dose-dependent precision of the phase extraction of charge transfer due to bonding for WS_2, we show in SFig. 6 the effect on the quantified S_2/W phase ratio for the IAM and DFT potentials using first just three pairs of sites. Since a ratio is calculated in this analysis, the relative errors between both atomic sites are summed for the total relative error. The uncertainties are sufficiently small to distinguish the IAM (no bonding) and DFT (bonding) simulations for doses of 5×10^4 e^-Å^{-2} and above for three measurements. The standard error of the mean decreases with the square root of the number of sites, $\sqrt{N}$. Thus for instance with 10 pairs of sites, the influence of charge transfer is significant compared to the uncertainty at doses at least as low as 5×10^3 e^-Å^{-2}.

S6. Phase shifts at defective sites

SFig. 7 shows a large area of the defective WS_2, with smaller regions highlighted on the right side. The green box shows a small region of pristine lattice, the purple box a region with several S vacancies, and the cyan box a full line defect. In all cases, the SSB shows a much clearer image of the defects than the ADF image; it is difficult to

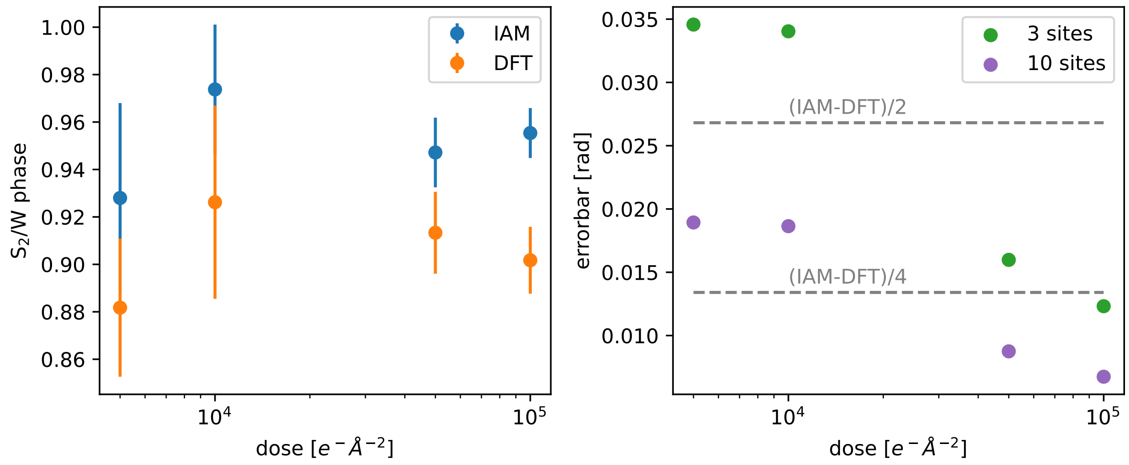

Supplementary Figure 6. Accuracy of phase extraction. Left: Ratio between the S_2 and W site phases at different doses. The error bar is calculated by the variation of phases as a function of Poisson noise. Right: Comparison of the ratios with different numbers of sampled sites. The horizontal dashed lines denote limits precision as fractions of the difference between IAM and DFT simulations.

even identify the S sites in the ADF signal. Besides this, the phase of W is also higher at sites with a higher defect density. This is due to both the contrast mechanism of SSB as well as charge transfer. All six different configurations discussed in the main text are present in this image.

SFig. 8a shows a simulation of the defective area that is used for the analysis in Fig. 3 of the main text containing several monovacancies. SFig. 8b shows another example of a small region of the experimental SSB image of a defective region. The experimental phases from all the sites in SFig. 8b are extracted and shown in the corresponding histograms on the right. For this specific region, the S vacancies, the pristine sites (S_2 and W) and the W close to the S vacancies (W@S_{vac}) are identified manually. As one can see, the W@S_{vac} sites have a higher phase than the corresponding pristine W. This is a result of the charge transfer at the defective sites, as discussed in the main text. The magnitude of the shift is related to the amount of charge transfer.

SFig. 9 shows SSB image simulations of structures with an increasing number of vacancies inserted around a W atom. Simulations based on both the DFT and IAM potentials as well as the difference between the corresponding SSB images are presented. The difference images show the result of the charge transfer, as the charge transfer is the only significant difference between the DFT and IAM images of a given model. The first three columns show one to three monovacancies (MVs) surrounding a W site. In the last three columns divacancies (DVs) are formed by removing the remaining S atoms from the MV sites, meaning there is a systematic increase of the

density of vacancies from left to right. The image contrast is kept constant, with a set range of displayed phase values for each row of images in the figure. It is clear that the difference between the DFT and IAM increases with the vacancy density, especially at the W site around which the vacancies are introduced.

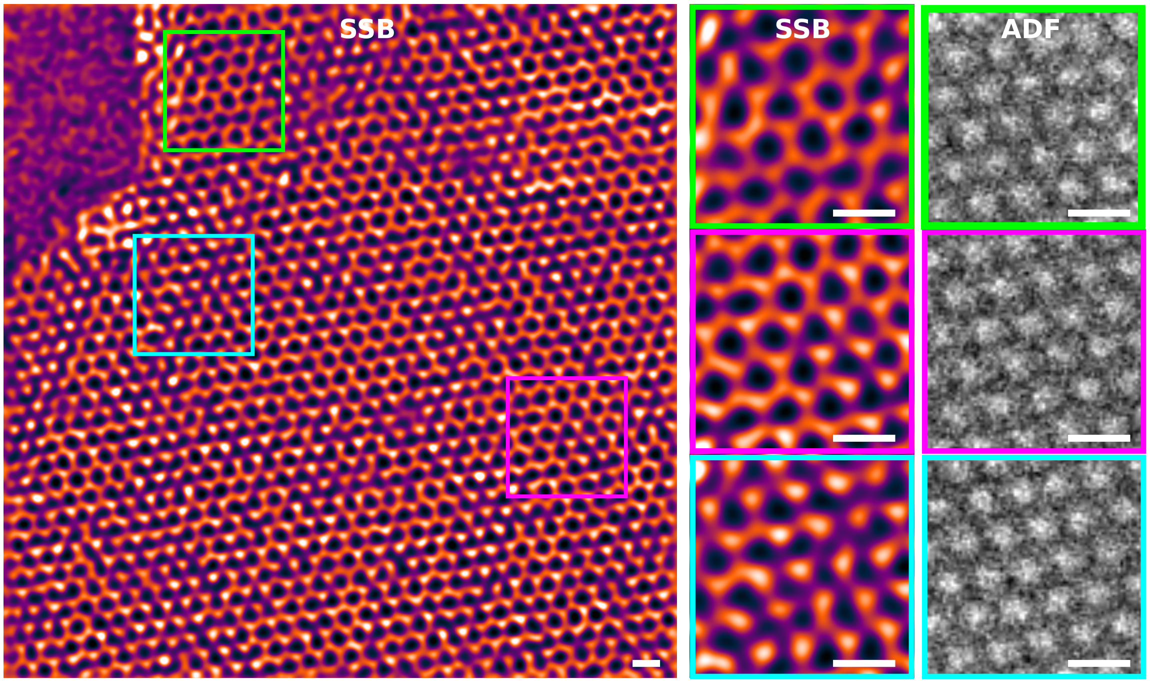

Supplementary Figure 7. Experimental data from a defected region of WS_2. Regions of different vacancy configurations and defect densities are highlighted on the right. The green box shows a pristine region, the purple box mono vacancies and the blue box a region with divacancies. These atomic structure of the defect sites can be only identified in the SSB images.

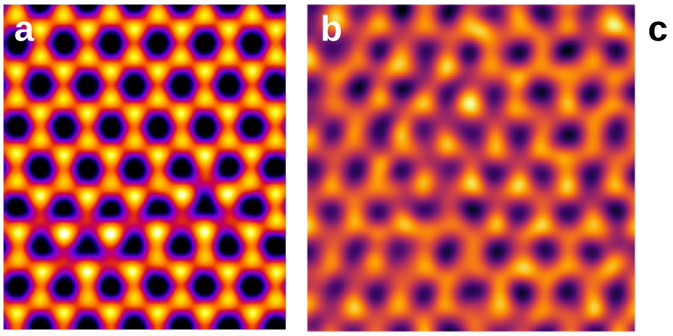

Supplementary Figure 8. Analysis of phase cross sections at a defective area. a) Simulated area that is analysed for Fig. 3c in the main manuscript. b) Experimental SSB image of a small defective area. c) Extracted phases of (b).

SFig. 10 shows the IAM and DFT projected potentials and their differences for the same defect configurations as in SFig. 9. The same trend as in the SSB images is observed: as the vacancy density increases (left to right), the DFT potential increases at the W site next to the S vacancies compared to the IAM potential. This demonstrates that the phases are directly related to the potentials and the changes in them are caused by bonding and charge transfer.

To elucidate the origin of the increase of the phase at the W site that occurs with the increase in the density of vacancies around it, we calculated the 3D pseudo valence electron density for pristine WS_2, V_{3S}, V_{3S_2}, and an isolated W atom. From the 3D volume, we calculated top and side view 2D electron density maps of a single slice of 0.07 Å thickness along the WS_2 sheet at the position of the W atom adjacent to the vacancies, as shown in SFig. 11. In this thin slice centered on the W layer of atoms, the electron density of the S atoms is not visibly apparent as viewed from the top. In both top and side view electron density maps, the electron density can be seen to shift away from W site as vacancies are introduced with the V_{3S_2} configuration having the lowest density of valence electrons around the $W@S_{vac}$ and the greatest increase between it and the neighboring W atoms. Note that the completely isolated W atom has the greatest concentration of valence electron density around it. Therefore it is not simply the absence of S atoms around the a W atom that reduces the concentration of valence electrons around a $W@S_{vac}$, but rather the charge transfer involved in a change in the bonding as the vacancies are introduced to the pristine structure. Compared to the isolated W atom, the electron density shifts away from the W atom when it is placed in the pristine structure. The electron density then shifts further away from the W atom as vacancies are introduced around it and the bonds essentially extend across longer distances. This is in agreement with the phase contrast we observe at different vacancy densities: the lower screening coming from the reduction of the electron density at a W site as the amount of vacancies increases also increases the phase at the $W@S_{vac}$. Thus the phase of the $W@S_{vac}$ is greatest for the V_{3S_2} configuration.

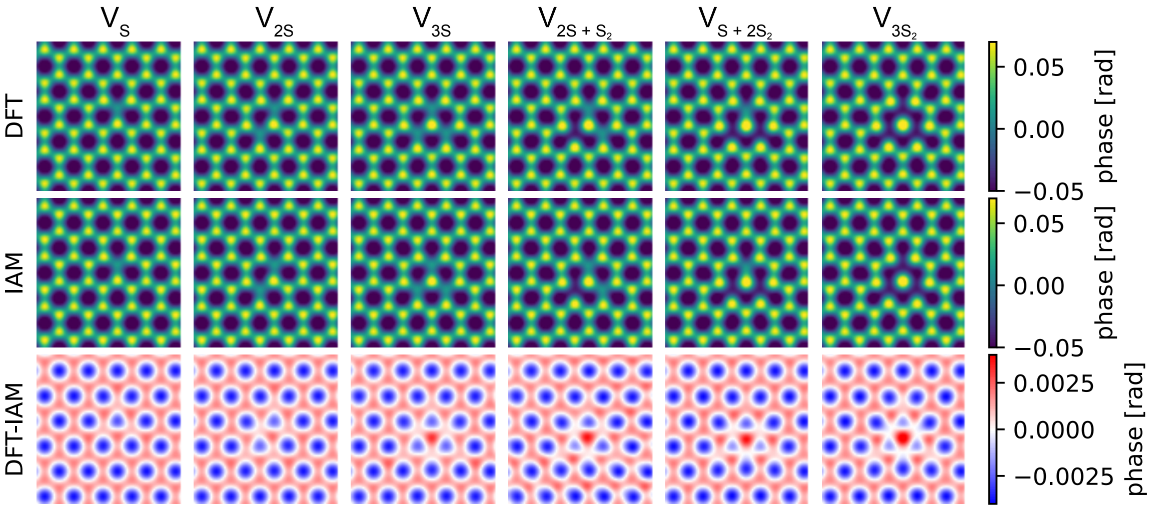

Supplementary Figure 9. SSB ptychography images simulated based on DFT and IAM potentials and their difference for different vacancy configurations. As the density of vacancies increases (left to right) more charge is transferred, resulting in greater contrast in the DFT–IAM image. The configurations correspond to a single monovacancy (MV), two MVs, three MVs, a single divacancy (DV), two DVs and three DVs.

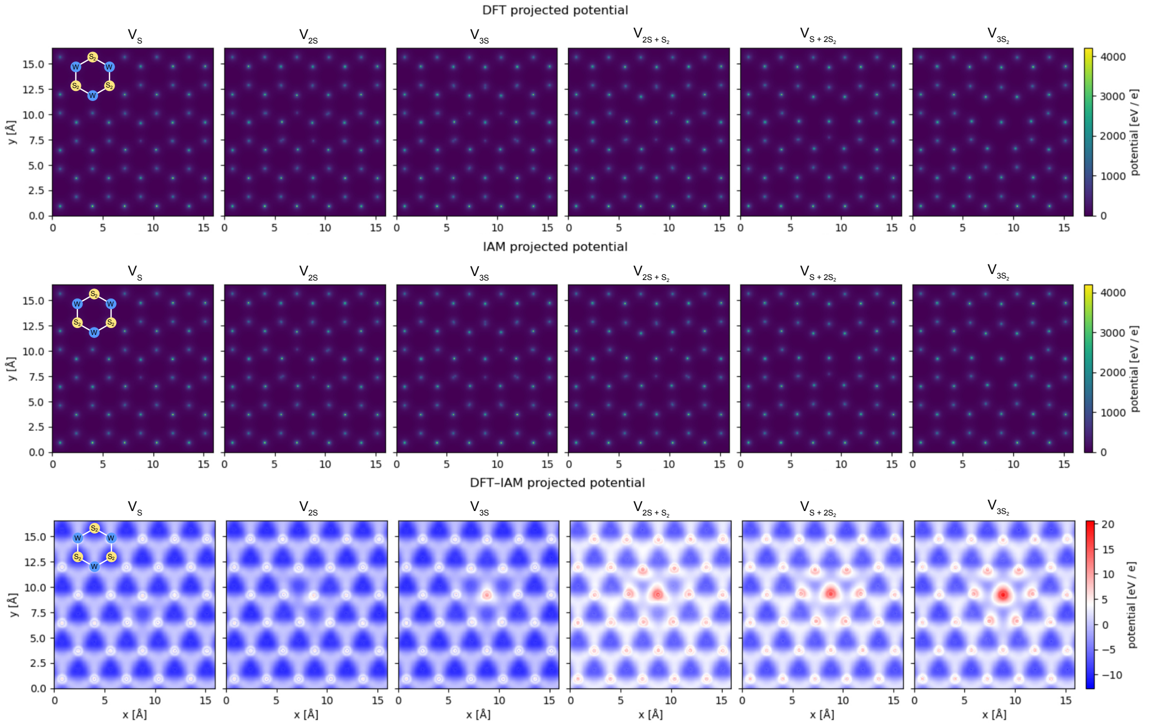

Supplementary Figure 10. Projected DFT and IAM potentials and their difference for the vacancy configurations shown in SFig. 9. The density of defects (vacancies) increases left to right.

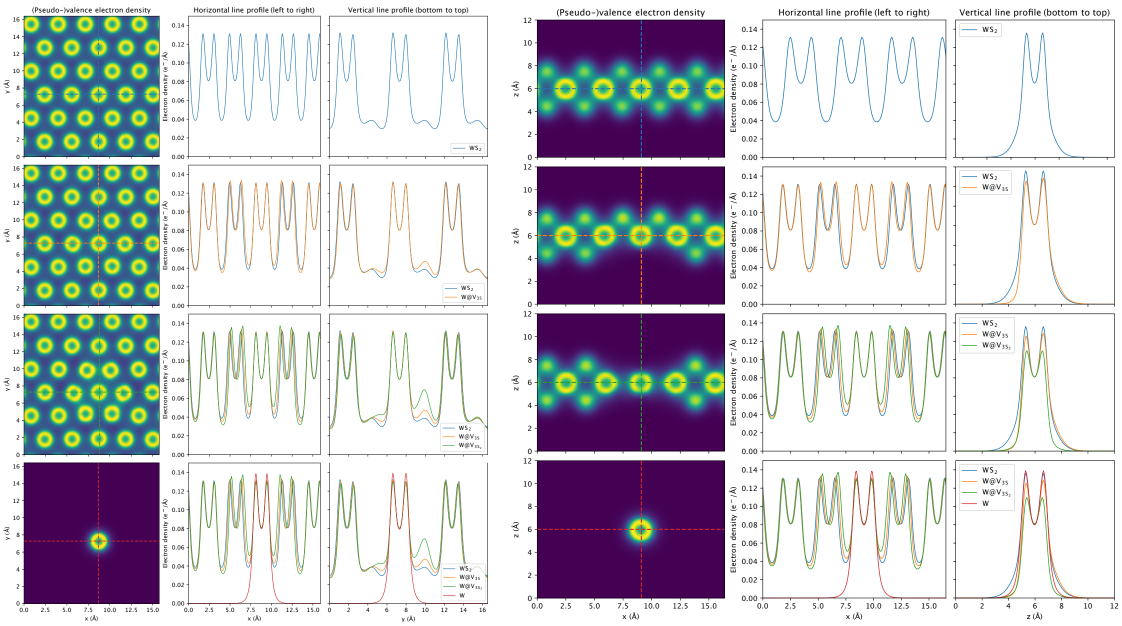

Supplementary Figure 11. Pseudo valence electron density of (defective) WS_2 in top view (left) and side view (right). From the 3D pseudo valence densities, the maps (first column) show a 2D slice (0.07 Å thickness) at the height of the W atoms (fourth column). The density of defects is increased from top to bottom. The valence electron density is decreasing at the W site close to the defect, explaining the phase increase. (The asymmetry visible in the vertical line profiles in the third column is due to the numerical sampling of the line profile over the pseudo valence electron density.)
